# When a Tooth Pulls the Heart Strings: Takotsubo Cardiomyopathy Post-dental Extraction

**DOI:** 10.7759/cureus.42044

**Published:** 2023-07-17

**Authors:** Muhammad Adil Afzal, Sacide S Ozgur, Yezin Shamoon, Rachel Abboud, Fayez Shamoon

**Affiliations:** 1 Internal Medicine, St. Joseph's University Medical Center, Paterson, USA; 2 Cardiology, St. Joseph's University Medical Center, Paterson, USA

**Keywords:** dental procedures, dental extraction, apical ballooning, stress-induced cardiomyopathy, takotsubo cardiomyopathy

## Abstract

Takotsubo cardiomyopathy, also known as stress-induced cardiomyopathy, is a temporary left ventricular dysfunction caused by a catecholamine surge under severe stress. It's characterized by chest pain, non-specific ECG changes, and left ventricular apical ballooning observed during catheterization. We present a case of a 59-year-old postmenopausal female with a past medical history of asthma who arrived at the ED complaining of chest pain following dental extraction. The patient's abnormal ECG findings and elevated cardiac enzymes required cardiac catheterization, which revealed normal coronary vasculature but demonstrated left ventricular apical ballooning. Transthoracic echocardiogram (TTE) showed septal left ventricular hypertrophy, decreased ejection fraction (EF), and akinetic segments consistent with takotsubo cardiomyopathy. Secondary takotsubo cardiomyopathy induced by stress in the setting of dental procedures like a tooth extraction for a periapical dental abscess is rarely described in the literature. Our case serves as a reminder of the potential for stress-induced cardiomyopathy in postmenopausal women, especially those with undiagnosed underlying anxiety disorders, even following minimally invasive procedures.

## Introduction

Takotsubo cardiomyopathy, also known as stress-induced cardiomyopathy or broken heart syndrome, is a unique form of transient left ventricular dysfunction, first described in 1990 by Sato et al. [1], which mimics acute myocardial infarction (AMI) [2] but lacks significant coronary artery disease. It is characterized by the development of apical ballooning of the left ventricle (LV), myocardial stunning, typically occurring in response to catecholamine surges that are produced as a result of emotional or physical stressors [3]. Takotsubo cardiomyopathy is most commonly seen in postmenopausal women and commonly presents with chest pain and dyspnea [4]. Although the exact prevalence of takotsubo cardiomyopathy is difficult to ascertain because of its close resemblance with myocardial infarction, literature reports it to be 1-0-2.5% [4]. The diagnostic criteria of takotsubo cardiomyopathy include a temporal relationship with a stressor, temporary left ventricular segment akinesia or dyskinesia, non-specific ECG changes, and absent coronary artery disease, myocarditis, or pheochromocytoma [2].

Although the link between stresses, including emotional trauma, serious sickness, and surgical procedures and the development of takotsubo cardiomyopathy has been well-documented, it is still extremely uncommon and underreported in the literature [5,6].

Understanding the link between dental treatments and the development of takotsubo cardiomyopathy is critical for early detection, correct diagnosis, and adequate therapy of this disorder. The case report points out the need for more research into the underlying processes and potential preventative measures and adds to the scant literature that is currently available on takotsubo cardiomyopathy connected to dental operations.

## Case presentation

A 59-year-old female with a past medical history of asthma presented to the ED with chest pain. The patient had been experiencing right maxillary pain and swelling, associated with subjective fever and chills, three days prior to the tooth extraction procedure and was diagnosed with a periapical dental abscess. The procedure was performed under local anesthesia (lidocaine), and the patient was prescribed amoxicillin and metronidazole and sent home. One-hour post-extraction, the patient developed a sudden onset of sharp left-sided chest pain radiating to her back, accompanied by nausea and dizziness. The chest pain persisted until the patient received a sublingual nitrate in the ambulance. She denied any shortness of breath, palpitations, leg swelling, orthopnea, paroxysmal nocturnal dyspnea, cough, wheezing, abdominal pain, vomiting, headache, blurry vision, numbness, or weakness.

**Figure 1:**
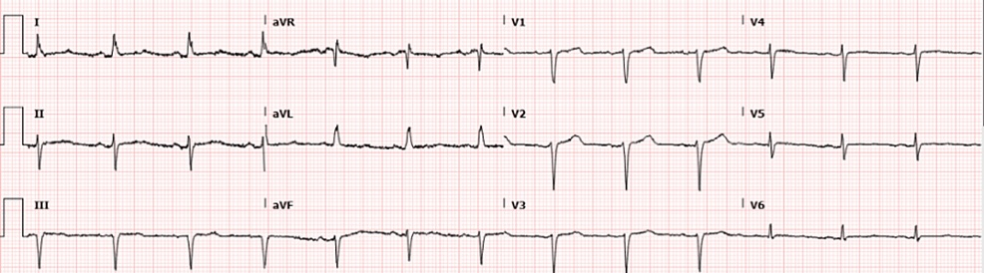
ECG showing normal sinus rhythm with normal axis. Diffuse ST segment flattening in all leads

In the ED, the patient was afebrile and saturating at 96% on room air. Her vital signs were significant for a blood pressure of 167/106 mmHg and a heart rate of 88 per minute. The cardiovascular and respiratory exam was normal, no focal neurologic deficits were detected, and the remainder of the physical exam was unremarkable. The ECG showed a normal sinus rhythm with a diffuse ST segment flattening (Figure 1).

**Figure 2:**
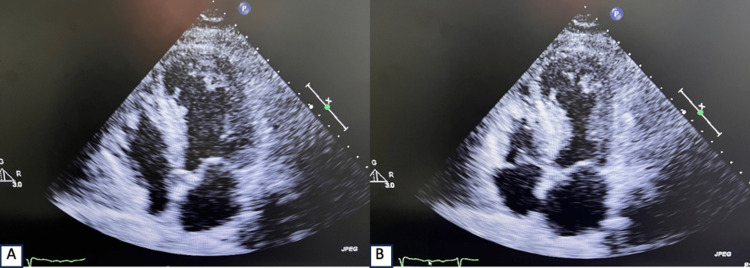
Panel A and B: TTE apical four-chamber view showing basal hyperkinesis with apical ballooning

The chest X-ray was negative for any abnormalities. Lab results showed leukocytosis with a white blood cell count of 13x10^9^/L (reference range is 4.5-11x10^9^/L) and elevated troponin levels of 177 pg/ml (reference range is 3-17 pg/ml), which trended up to 1300 pg/ml. The patient was admitted to the cardiac intensive care unit with the diagnosis of non-ST segment elevation myocardial infarction (NSTEMI). The acute coronary syndrome (ACS) protocol was initiated; the patient was given 325 mg of aspirin and 300 mg of clopidogrel, and a heparin drip was started. The patient was also started on lisinopril 5 mg daily and metoprolol succinate 25 mg daily. The patient underwent a left heart catheterization the next day of admission, which showed no coronary occlusion and was significant for LV apical ballooning on LV gram. The transthoracic echocardiogram (TTE) showed a reduced ejection fraction (EF) of the LV of 30% to 35% with a normal LV cavity size, mildly increased LV wall thickness with septal left ventricular hypertrophy, and basal hyperkinesis with apical ballooning (Figure 2, Video 1).

**Video 1:** TTE apical four-chamber view demonstrating basal hyperkinesis with apical ballooning

The LV wall motion score was significant for the akinetic apical septal segment, apical lateral segment, and apex. The global right ventricular systolic function was normal, and the left atrium size was normal.

The patient was diagnosed with takotsubo cardiomyopathy. The patient's chest pain resolved, blood pressure optimized, and troponin downtrended. She remained hemodynamically stable on the telemetry floor and was discharged to home with lisinopril 5 mg daily, metoprolol succinate 25 mg daily, and atorvastatin 40 mg daily. At the two-week cardiology follow-up, the patient denied any complaints. Her blood pressure was better controlled, and titration of goal-directed medical therapy was initiated, including lisinopril 20 mg daily, metoprolol succinate 25 mg daily, empagliflozin 10 mg daily, and spironolactone 25 mg daily. There is a plan for repeat TTE at the three-month follow-up visit.

## Discussion

Takotsubo cardiomyopathy is a unique form of cardiomyopathy characterized by transient left ventricular dysfunction. Catecholamine excess and abnormal corticosteroid hormonal homeostasis have been reported to produce cardiac stunning-like findings in patients [7,8]. It is, therefore, believed to result from a surge of catecholamines in response to a physical or emotional stressor, leading to distinctive cardiac changes, including apical ballooning, regional wall motion abnormalities, elevated troponin levels, and reduced EF on echocardiography in the absence of coronary artery disease [3,9]. Although reversible in nature, takotsubo cardiomyopathy can present with clinical signs similar to myocardial infarction and requires prompt recognition and urgent management due to its potential for complications and even fatal outcomes [3,9,10].

Our case presents the clinical scenario of a known asthmatic woman with an anxiety disorder who experienced sudden onset chest pain and breathlessness resembling angina or acute myocardial infarction following a tooth extraction procedure. Her elevated troponin levels, reduced left ventricular EF on echocardiography, and non-specific ECG findings initially raised suspicion for NSTEMI. However, the subsequent evaluation revealed disease-free coronary arteries. Remarkably, the patient demonstrated a decline in troponin levels and improvement in echocardiographic findings within the next two days, ultimately confirming the diagnosis of takotsubo cardiomyopathy. This case underscores the important observation that takotsubo cardiomyopathy can present with clinical features similar to myocardial infarction.

One important risk factor that could have led to the development of takotsubo in this patient could be her underlying anxiety disorder that made her prone to an intense stress response. While rare, it is noteworthy that dental procedures have been implicated in the development of takotsubo cardiomyopathy, both in patients with and without a dental phobia, as demonstrated by Higuchi et al. [5], Bleser et al. [10], and Takuma et al. [6]. The possible cause of the intense stress response observed in patients undergoing even minimally invasive oral surgeries, such as the tooth extraction in our case, could be attributed to the close proximity of the procedure to the face and oral cavity. Higuchi et al. postulated that the use of restraints during dental procedures could be an escalating factor leading to the development of takotsubo [5]. However, the precise etiology and underlying mechanisms of this heightened stress response are not yet fully understood. Further research is needed to elucidate the factors contributing to the intense physiological and psychological stress experienced by some individuals during dental procedures, particularly in relation to the development of takotsubo cardiomyopathy. Investigating potential triggers and examining the pathophysiological pathways involved will help improve our understanding of the condition and guide future preventive and management strategies.

Takotsubo cardiomyopathy, due to its variable presentation and close similarities with AMI, is a diagnosis of exclusion. Therefore, it is essential to rule out other potential causes of chest pain, elevated troponin levels, ECG changes, and ventricular akinesia before arriving at a definitive diagnosis of takotsubo cardiomyopathy [9]. In our case, comprehensive evaluations were performed to exclude alternative differential diagnoses. Notably, myocarditis and significant coronary artery disease were ruled out, further supporting the diagnosis of takotsubo cardiomyopathy [3]. It is noteworthy that takotsubo cardiomyopathy predominantly affects postmenopausal women, with estrogen deficiency being a recognized risk factor for its development [3]. This demographic characteristic aligns with our patient's profile, as she is a postmenopausal woman.

Since takotsubo is clinically indistinguishable from myocardial infarction during its initial stages and is almost equally, if not more fatal, therefore, ACS protocol should be started as soon as possible, with the initial therapy comprising of oxygen inhalation, beta-blockers, aspirin, and intravenous heparin [11]. Beta-blocker may be additionally beneficial in the setting of the catecholamine surge as the causative factor of takotsubo, though they should be stopped if coronary vasospasm is suspected. Aspirin therapy can be stopped if coronary artery disease is found to be non-existent, as was done in our patient's case [11]. Angiotensin-converting enzyme inhibitors may also be used to minimize ventricular wall abnormality [11]. We used lisinopril in our patient both to enhance recovery of the myocardial wall abnormality and to control her blood pressure.

## Conclusions

Our case report underscores the significance of maintaining vigilance during acutely stressful procedures, such as dental interventions, and raises awareness regarding the possibility of takotsubo cardiomyopathy. While this condition can be potentially fatal, it is crucial to recognize that it is reversible, and fatalities can be avoided with prompt diagnosis and appropriate management. Moreover, our findings highlight the importance of maintaining a low threshold for considering the development of takotsubo cardiomyopathy in patients with anxiety or panic disorders. By understanding the unique challenges and potential triggers associated with takotsubo cardiomyopathy, healthcare providers can enhance patient care, improve outcomes, and promote preventive measures.
